# Genome-Wide Analysis of Small RNA and Novel MicroRNA Discovery during Fiber and Seed Initial Development in *Gossypium hirsutum*. L

**DOI:** 10.1371/journal.pone.0069743

**Published:** 2013-07-29

**Authors:** Hua Zhang, Qun Wan, Wenxue Ye, Yuanda Lv, Huaitong Wu, Tianzhen Zhang

**Affiliations:** National Key Laboratory of Crop Genetics and Germplasm Enhancement, Hybrid Cotton R & D Engineering Center/the Ministre of Education, Cotton Research Institute, Nanjing Agricultural University, Nanjing, China; East Carolina University, United States of America

## Abstract

Cotton is the source of the most important, renewable natural textile fiber and oil in the world. MicroRNAs (miRNAs) are endogenous, non-coding, approximately 18–24 nucleotides long RNAs and function in the negative regulation of their target genes. Two mostly overlapping libraries of small RNA molecules were constructed and sequenced, and served as repetition sets of data to identify miRNAs involved in fiber initiation and seed development. The D genome sequence of *Gossypium raimondii* was used in conjunction with EST sequences to predict miRNA precursors. Overall, 93 new miRNA precursors were identified, of which 28 belonged to 10 known families and the other 65 were considered to be novel miRNAs. Seven hundred EST sequences were proposed to be candidate target genes which involved in the regulation of a diverse group of genes with diverse functions and transcription factors. Some of the novel miRNAs and candidate target genes were validated by the Northern blot and rapid amplification of 5′ cDNA ends (5′ RACE).

## Introduction

MicroRNAs (miRNAs) are endogenous, non-coding RNA molecules that are approximately 18–24 nucleotides in length. In plants cell, Dicer like1 (DCL1), orchestrates both catalyze processes in the nucleus, and this results in ∼21 nucleotide mature miRNA/miRNA* (the complementary strand of miRNA) duplex after which they are exported into the cytoplasm [1]–[5]. Increasing evidence has demonstrated that plant miRNAs negatively regulate their target genes, which have functions in a wide range of developmental processes [6] by mRNA cleavage or translation repression [7]. A substantially increasing number of plant miRNAs populate a number of databases, such as miRBase [8], and sets of precursors and mature miRNAs have been discovered in many different plant genomes. From 2007 onwards, almost the entire growth of miRBase has been driven by deep sequencing experiments [8].

Cotton is the source of the most important renewable, natural textile fiber in the world and is of significant importance in the textile industry. Cotton ‘fibers’ are trichomes derived from epidermal cells of the developing seed [9]. These trichomes share many similarities with those found on *Arabidopsis thaliana* leaves and which could serve as a model for elucidating the genetic mechanisms that control cotton fiber and seed development [10]. In *Arabidopsis* and maize (*Zea mays* L.), it have been validated miR172 which targets Apetala 2 (AP2) transcription factor, together with miR156 whose targets are a series of Squamosa promoter binding protein-like (SPL) transcription factors, regulate developmental transitions and guide various aspects of reproductive development in complementary patterns [11]–[14]. In *Arabidopsis*, miR156 temporally controls trichome distribution during flowering *via* its target *SPL*9, which define an endogenous flowering pathway and establish a direct link between developmental programming and trichome distribution [15]. Plants that overexpressed miR156 developed ectopic trichomes on the stem and floral organs [15]. MiR165/166 targets the class III Homeodomain leucine zipper (HD-ZIP III) family of transcription factors that were investigated widely because of their great functions in the regulation of organ polarity and morphogenesis [6]. The abnormal distribution of trichomes on two mutants of the *Incurvata* (*Icu*) gene, which encodes ATHB15, is probably due to a single nucleotide mutation within a miRNA-mRNA complementarily area [16]. Auxin plays a critical role in cotton fiber initiation and the initiated fiber cells may be the site of indole-3-acetic acid (IAA) accumulation [17] and, moreover, promote seed development [18]. Both miR167 and miR160 could target *AUXIN RESPONSE FACTOR* (*ARF*) mRNAs. Nevertheless, miR160 and miR167 targets appear to have opposite roles with respect to controlling the expression of the auxin homeostatic enzyme GH3 [6]. It has also been documented that miR167 is essential for fertility of both ovules and anthers [19]. Mutations in the miR167 target sites of ARF6 or ARF8 are responsible for the ectopic expression of these genes in ovules, which results in the arrested development of integuments and ovule sterility [19]. During early embryogenesis, miRNAs repress expressing of *LEAFY COTYLEDON*2 and *FUSCA*3, while *ARABIDOPSIS 6B-INTERACTING PROTEIN1- LIKE1* (*ASIL1*), *ASIL2* and the histone deacetylase *HDA6/SIL1* act as downstream components of miRNAs to repress maturation [20].

Up to now, 43 cotton miRNA genes are currently registered in miRBase (Release 19.0, August 2012), which encode 45 mature miRNAs including two miRNA* sequences. On the other hand, 348 miRNA were proposed based on genome of *G. raimondii* released by Cotton Genome Project (CGP) [21]. To further understand the various roles miRNAs play in cotton young ovules during fiber cell initiation, we have constructed two small RNA libraries from cotton ovules at critical times of fiber cell development initiation and 33 million clean reads were achieved in total. Besides EST sequences released on NCBI, we aligned small RNAs with D genome sequence of *G. raimondii* [21], [22], and candidate precursors were predicted. After strict filtration, 93 new miRNA precursors were identified, 28 of which produce mature miRNAs that belong to 10 miRNA families and another 65 were suggested as novel miRNAs of cotton. A total of 693 EST sequences were obtained and identified as candidate target genes involved in diverse functions besides transcription factors.

## Materials and Methods

### Plant materials preparation and total RNA isolation

The Upland cotton, *Gossypium hirsutum* acc. Texas Marker-1 (TM-1), was grown at Jiangpu Breeding Station, Nanjing (JBS/NAU) in 2010. Flowers were tied up one day before anthesis (−1 DPA) to ensure self-pollination. The −3 DPA and −1 DPA flowers were estimated based on flower size and shape. Flowers and bolls were harvested at around 4 pm after pollination and stored in ice. Intraday, ovules were dissected carefully from each boll, frozen in liquid nitrogen and stored at −70°C. Total RNA was extracted using the CTAB method [23]. We pooled the total RNAs from −3, −1, 0 and 1 DPA ovules in an equal fraction ratio, and named it TM-1L-A library. The same procedure was done to −1, 0, 1, 3 and 5 DPA samples, which was named TM-1L-B.

### Small RNA sequencing and annotation

Solexa sequencing technology was employed to sequence the small RNAs from pooled cotton ovules samples. After summarizing the length distribution of clean reads, the small RNA reads were mapped to cotton ESTs on NCBI and D genome sequence to analyze their expression and distribution on the reference sequences. The standard bioinformatics analysis annotated the clean reads with rRNA, scRNA, snoRNA, snRNA and tRNA from GenBank and Rfam to delete matched reads. The remains of mapped sequences were used to predict miRNA precursors based on common criteria [24].

### Qualifications for the prediction of new miRNAs

The prediction software Mireap (http://sourceforge.net/projects/mireap) developed by BGI (Beijing Genome Institute at Shenzhen, China) was used to predict new miRNAs by exploring their secondary structure, the Dicer cleavage site and the minimum free energy (MFE) of the small RNA reads, which could be mapped to genome or EST sequences. The primary criterion was that small RNA was precisely excised from the stem of a stem-loop precursor [24]. Specifically, only dominant, mature sequences that were counted more than 10 times and located within the stem region of the stem-loop structure and ranged between 20–24 nt with a maximum MFE of −20 kcal·mol^−1^ were considered. A maximum of four unpaired nucleotides between the miRNA and miRNA* were allowed. To enhance the reliability of the results and support a consistent processing of miRNAs at the 5′-end, we only preserve the one that the majority of reads had an identical 5′-end. The selected sequences were then folded into a secondary structure using an RNA-folding program mFold 3.2 [25]. When a perfect stem-loop structure was formed, the small RNA sequence sat at one arm of the stem and asymmetric bulges were minimal in size (one base at most) and infrequent (typically one or less) within the miRNA/miRNA* duplex. These small RNA structures that formed were proposed to be new cotton miRNAs.

### Northern blotting of mature miRNAs

A total of 10 µg RNA was resolved on 15% polyacrylamide (29∶1) gels containing 8 M Urea and 2×SSC (0.3M NaCl, 30 mM Sodium Citrate, pH 7) under denaturing conditions. RNA was blotted onto a Hybond-N^+^ nylon membrane (Roche, Basel, Switzerland) using a Trans-Blot SD Semi-Dry lectrophoretic Transfer Cell (Bio-Rad, Hercules, CA, USA). Then the membrane was cross-linked by UV at 1200 mJ in Stratagene UV Stratalinker 1800 for 60 sec. Antisense oligonucleotide (20 pmol) for each miRNA (Table S5) was 5′-end labeled (γ-^32^P-ATP) by T4 polynucleotide kinase (NEB, Ipswich, MA, USA) to detect mature miRNAs. U6 was used as the loading control. Hybridization was performed in 5×SSC, 20 mM Na_2_HPO4, 3×Denhardt's solution (1×Denhardt's: 0.02% Ficoll, 0.02% polyvinylpyrrolidone, and 0.02% BSA), and 0.7% SDS with competitor Herring sperm DNA (0.1 mg/ml, Sigma-Aldrich, St. Louis, MO, USA). Prehybridization (2 h) and hybridization (overnight) were performed at 37°C. After hybridization, the membrane was washed twice with 2×SSC and 0.2×SDS at 37°C for 10 min. Hybridized membranes were exposed to a storage phosphor screen (GE Healthcare, Buckinghamshire, UK) for 1–4 days and the screens were scanned using a Storm 825 phosphoimager (GE Healthcare, Buckinghamshire, UK).

### Target genes prediction

The targets of miRNAs were predicted by the web tool psRNATarget [26] using the *Gossypium* (cotton) DFCI Gene Index (CGI) Release 11 (http://compbio.dfci.harvard.edu/index.html) as the sequence library for the targets search. No more than three mismatches between miRNAs and targets were allowed, especially no mismatches were allowed within the maximum expectation region. Gene Ontology (GO) annotation was used to infer target genes function description by Blast2GO [27].

### RNA ligase-mediated 5′ RACE

To validate the cleavage sites of target genes, we used RNA ligation-mediated (RLM) rapid amplification of 5′ complementary DNA ends (5′ RACE) using a GeneRacer™ kit (Invitrogen, Carlsbad, CA, USA). Four mg RNAs from 3DPA ovule was ligated to a 5′ RACE RNA adapter without calf intestine alkaline phosphatase treatment. cDNAs were transcribed with reverse transcriptase by the GeneRacer™ Oligo dT primer and used as the template for PCR amplification with GeneRacer™ 5′ Primer, GeneRacer™ 5′Nested Primer and two gene-specific reverse primers for each RACE. In each case, the PCR products were gel purified, cloned, and sequenced. PCR primers are shown in Table S5.

## Results

### Distribution of small RNA in cotton fiber cells

**Figure 1:**
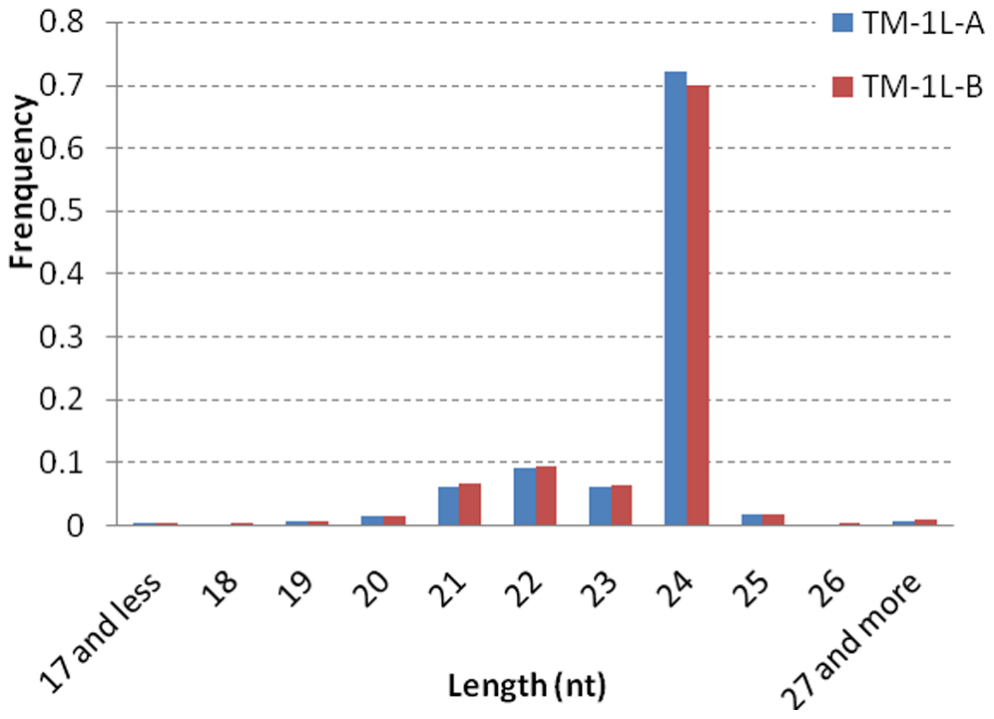
Sequence length distribution of cotton small RNA libraries of TM-1L-A and TM-1L-B. 24-nucleotides reads were of significant greater proportion (∼70%) than others.

After the deep sequencing of the two libraries, more than thirty-three million reads were obtained, 17,027,097 and 16,441,172 reads for TM-1L-A and TM-1L-B (Table 1), respectively. After the deletion of low quality reads and several kinds of contaminant tags, 32,665,490 (97.6%) clean reads were obtained and the distinct reads for further analysis were 8,205,472 and 7,834,823 for TM-1L-A and TM-1L-B, respectively. Among all of the clean reads, 24 nucleotide sequences were found in significantly greater numbers than the others, and accounted for 72% and 70% of the total reads (Figure 1). The 22 nt reads were second (9.2% and 9.57%), and followed by 21 nt (6.38% and 6.81%) and 23 nt (6.25% and 6.51%) reads.

**Table 1:** Statistics of small RNA sequence reads.

	TM-1L-A				TM-1L-B			
	Distinct reads		All reads		Distinct reads		All reads	
Total:	8205472		16622462		7834823		16043028	
match genome	5042549	61.45%	10842192	65.23%	4844473	61.83%	10590216	66.01%
rRNA	48298	0.59%	465537	2.80%	56026	0.72%	673245	4.20%
siRNA	269364	3.30%	1108447	6.67%	247443	3.16%	992828	6.19%
snRNA	947	0.01%	1772	0.01%	1090	0.01%	2075	0.01%
snoRNA	669	0.01%	1378	0.01%	716	0.01%	1534	0.01%
tRNA	4051	0.05%	34052	0.20%	5577	0.07%	54078	0.34%
unannotation	7863905	95.84%	14284880	85.94%	7506820	95.81%	13595072	84.74%

The small RNA reads were mapped to the genome and about more than sixty percent of distinct reads or 65% and 66% of the total reads were mapped to the *Gossypium* genome (DOE Joint Genome Institute: Cotton D V1.0). Approximately 4% of the distinct reads matched non-coding rRNA, scRNA, snoRNA, snRNA and tRNA, which accounted for 9.69% and 10.7% of TM-1L-A and TM-1L-B, respectively (Table 1). The majority of the remaining reads were documented as unannotated reads for further analysis.

### miRNAs expressed differently between each other during fiber cell initiation

There were 33 miRNA families were identified in TM-1L-A and TM-1L-B. The miR166 was the mostly accumulated miRNAs in the young ovules and fibers of cotton with a total of 153,922 reads detected between the two libraries, [78,159 miRNAs in TM-1L-A and 75,163 miRNAs in TM-1L-B (Table 2)]. There was considerable expression levels diversity between the families probably due to that the cotton ovule is a highly differentiated organ and genes are expressed dynamically during development. For example, miR166 and miR172 were both sequenced more than seventy thousand (∼42%) and sixty thousand (∼35%) times between both of the two libraries, respectively. In contrast to the most abundant miRNAs, miR828, miR475 and miR1023 (Table 2) were detected less than 10 times, which confirmed that miRNA expression maybe developmental and/or tissue specific [28]. Members of the same family did not express equally either. For example, among the miR156/157 members, the mature sequence miR157a/b (UUGACAGAAGAUAGAGAGCAC) was accumulated much more than miR156d (UGACAGAAGAGAGUGAGCAC). The two libraries TM-1L-A and TM-1L-B mostly overlapped and the majority of the families were expressed in equal levels between the libraries, but there were some exceptions (Table 2). Six miRNAs miR160, miR394, miR397, miR398, miR482 and miR2111 were significantly upregulated in TM-1L-B compared to TM-1L-A, which suggested that miRNAs expression level were constantly changing during early cotton fiber and ovule development.

**Table 2:** Conserved miRNA families expression in cotton.

Family	Mature sequence	Total reads	TM-1L-A		TM-1L-B		Fold
			Raw reads	RPM reads	Raw reads	RPM reads	
miR156/157	UUGACAGAAGAUAGAGAGCAC	8374	4743	285.34	3631	226.33	1.26
miR159/319	UUUGGAUUGAAGGGAGCUCUA	5066	3235	194.62	1831	114.13	1.71
miR160	UGCCUGGCUCCCUGUAUGCCA	1383	229	13.78	1154	71.93	0.19
miR162	UCGAUAAACCUCUGCAUCCAG	2340	1315	79.11	1025	63.89	1.24
miR164	UGGAGAAGCAGGGCACGUGCA	10285	4189	252.01	6096	379.98	0.66
miR166	UCGGACCAGGCUUCAUUCCCC	153922	78159	4702.01	75763	4722.49	1.00
miR167	UGAAGCUGCCAGCAUGAUCU	13953	7691	462.69	6262	390.33	1.19
miR168	UCGCUUGGUGCAGGUCGGGAA	790	374	22.50	416	25.93	0.87
miR169	CAGCCAAGGAUGAUUUGCCGG	1096	465	27.97	631	39.33	0.71
miR171	UGAUUGAGCCGUGCCAAUAUC	1279	618	37.18	661	41.20	0.90
miR172	AGAAUCCUGAUGAUGCUGCAG	130236	66013	3971.31	64223	4003.17	0.99
miR390	AAGCUCAGGAGGGAUAGCGCC	5370	2839	170.79	2531	157.76	1.08
miR393	UCCAAAGGGAUCGCAUUGAUC	99	54	3.25	45	2.80	1.16
miR394	UUGGCAUUCUGUCCACCUCC	135	10	0.60	125	7.79	0.08
miR395	CUGAAGUGUUUGGGGGAACUC	21	10	0.60	11	0.69	0.88
miR396	UUCCACAGCUUUCUUGAACUU	703	307	18.47	396	24.68	0.75
miR397	UCAUUGAGUGCAGCGUUGAUG	163	34	2.05	129	8.04	0.25
miR398	UUCUCAGGUCACCCCUUUGGG	791	167	10.05	624	38.90	0.26
miR399	UGCCAAAGGAGAUUUGCCCGG	30	9	0.54	21	1.31	0.41
miR403	UUAGAUUCACGCACAAACUCG	7670	3799	228.55	3871	241.29	0.95
miR408	AUGCACUGCCUCUUCCCUGGC	30	6	0.36	24	1.50	0.24
miR475	UUACAAUUCCAUUGAUUAAACCGU	2	1	0.06	1	0.06	0.97
miR482	UUGCCUACUCCACCCAUGCCAC	1707	597	35.92	1110	69.19	0.52
miR530	UGCAUUUGCACCUGCACCUUC	123	46	2.77	77	4.80	0.58
miR535	UUGACAACGAGAGAGAGCACG	2878	1375	82.72	1503	93.69	0.88
miR827	UUAGAUGACCAUCAACAAACA	156	73	4.39	83	5.17	0.85
miR828	UCUUGCUCAAAUGAGUAUUCCA	8	2	0.12	6	0.37	0.32
miR1023	ACACUCUGUCAUAUCGUCUGC	4	1	0.06	3	0.19	0.32
miR2111	UAAUCUGCAUCCUGAGGUUUG	249	82	4.93	167	10.41	0.47
miR2947	UAUACCGUGCCCAUGACU	10178	4814	289.61	5364	334.35	0.87
miR2948	UGUGGGAGAGUUGGGCAAGAAU	284	160	9.63	124	7.73	1.25
miR2949	ACUUUUGAACUGGAUUUGCCGA	4719	2435	146.49	2284	142.37	1.03
miR3476	UGAACUGGGUUUGUUGGCUGC	3095	1534	92.28	1561	97.30	0.95

FoRPM, reads per million. Fold, fold change of TM-1L-A/TM-1L-B.

### Identification of new miRNAs in cotton

Thanks to deep-sequencing, a few new conserved and less conserved *MIRNA* genes were also identified. Overall, 28 *MIRNA* genes from 10 families survived a series of strict filtrations (Table S1 and Figure S1) and were named based on the common nomenclatural rules [8]: triliteral was prefixed according to the source of the sequence, while postfixes were used to distinguish the different precursors produce the same mature sequence. In addition, 65 precursors containing 43 small RNAs were identified but did not show enough similarity with any currently known miRNAs, and were labeled as novel miRNAs (Table S2 and Figure S2). Some of the novel MIRNAs produce remarkably similar even coincident mature sequence that we reckoned them probably evolved from the same ancestor, and different letters were suffixed for recognition. The precursor sequences were numbered in consecutive order from MIR7234 to MIR7276, and the mature sequences were named after their precursors as miR7234 to miR7276. The majority of the miRNAs (23 of the 43, 53.5%) have a uridine at 5′ terminal (Figure S3), showing a preference of AGO1 [29]. Differing from the miRNAs described as conserved families, none of the 43 novel miRNAs displayed unequal expression levels between the two libraries TM-1L-A and TM-1L-B (Table 3). MiRn7234 accumulated abundantly with more than twenty thousand reads. MiR7235, miR7236 and miR7237 were also detected in relatively high levels with more than one thousand reads in each library. However, the majority of the novel miRNAs (26, 60.5%) was detected in very low levels, and had less than 50 reads.

**Table 3:** Novel miRNAs identified in cotton.

miRNA ID	Mature miRNA sequence	Arm	G+C (%)	Total	TM-1L-A		TM-1L-B		Fold
					Raw reads	RPM reads	Raw reads	RPM reads	
miR7234	UUGGACAGAGUAAUCACGGUCG	5′	50.00%	425743	199505	12002.37	226238	14101.95	0.85
miR7235	UUUUGGAAGAAUUUCAGCUGG	5′	38.10%	10165	4656	280.11	5509	343.39	0.82
miR7236	ACAGCUUUAGAAAUCAUCCCU	5′	38.10%	5446	2924	175.91	2522	157.2	1.12
miR7237	UUACUUUAGAUGUCUCCUUCA	3′	33.33%	2414	1372	82.54	1042	64.95	1.27
miR7238	UCCAUAUUUCACUAUCUCUUA	3′	28.57%	1022	442	26.59	580	36.15	0.74
miR7239	UGAAUAUUGUUAAAGUAGAAA	3′	19.05%	506	240	14.44	266	16.58	0.87
miR7240	AAUAAGGGGCUUAGAAAGAUG	3′	38.10%	508	264	15.88	244	15.21	1.04
miR7241	GAUUUGGGGCAAAGACGGGAU	3′	52.38%	374	212	12.75	162	10.1	1.26
miR7242	AGGCUCUUUGUAGAAUCAGGAG	3′	45.45%	364	185	11.13	179	11.16	1
miR7243	UUCAGAAACCAUCCCUUCCUU	5′	42.86%	314	108	6.5	206	12.84	0.51
miR7244	UGGACUUAGCUGCCAAGUUUG	3′	47.62%	283	150	9.02	133	8.29	1.09
miR7245	UUCCAUGUCACAGAGAUGUUG	5′	42.86%	180	99	5.96	81	5.05	1.18
miR7246	GGAAUGUUGUCUGGACCGGGG	5′	61.90%	172	89	5.35	83	5.17	1.03
miR7247	UUGUGAUGUUUGUGAGGAACA	3′	38.10%	110	60	3.61	50	3.12	1.16
miR7248	UUGAAAAGAAUCCUUCAAACGU	3′	31.82%	103	54	3.25	49	3.05	1.07
miR7249	UCUGACAGUGCACUGAAAACG	3′	47.62%	101	43	2.59	58	3.62	0.72
miR7250	UCACAGGGAUCAAAAUUGGGA	3′	42.86%	68	33	1.99	35	2.18	0.91
miR7251	UAAGUGAAGAAAGAGGUAGGUU	5′	36.36%	154	76	4.57	78	4.86	0.94
miR7252	UGCUACUUGUAGUUAUGCAUG	3′	38.10%	90	48	2.89	42	2.62	1.1
miR7253	AUCAUGCGAUCCCUUCGGAAU	3′	47.62%	60	26	1.56	34	2.12	0.74
miR7254	AGCCCGAUUUUGGGCCUAGU	3′	55.00%	48	28	1.68	20	1.25	1.34
miR7255	AUGGAUGAAAUUUUUAACAGA	3′	23.81%	90	43	2.59	47	2.93	0.88
miR7256	CUUGGUAGAGCACAGGAGACA	3′	52.38%	48	20	1.2	28	1.75	0.69
miR7257	UGAUGGAGAUAGGUAUCUGCA	5′	42.86%	37	20	1.2	17	1.06	1.13
miR7258	AUAUGAUUUGUUAAGGCAAG	5′	30.00%	32	13	0.78	19	1.18	0.66
miR7259	UUAGAUCAAAGAGUAAACUAAUU	5′	21.74%	31	13	0.78	18	1.12	0.7
miR7260	UAGAAACUCGAUCGUCUUCU	3′	40.00%	46	16	0.96	30	1.87	0.51
miR7261	UCUGUCGCAGGGGAGAUGGCUG	5′	63.64%	37	7	0.42	30	1.87	0.22
miR7262	UAGACACUCUGGGCACAAUAG	3′	47.62%	28	12	0.72	16	1	0.72
miR7263	AUUGAUCUGUAUCGAUUAUCU	3′	28.57%	24	12	0.72	12	0.75	0.96
miR7264	ACUCUCUUCCAAAGGCUUCAAG	5′	45.45%	29	6	0.36	23	1.43	0.25
miR7265	CCACCGUCGAGGGUUCGAGAUCG	3′	65.22%	23	10	0.6	13	0.81	0.74
miR7266	AAUGGCAUUGAUGUAGCAGCU	5′	42.86%	21	1	0.06	20	1.25	0.05
miR7267	UUGUACGUUAGAUUAAAGAGC	5′	33.33%	24	7	0.42	17	1.06	0.4
miR7268	UUUAAAUCUAUAAAGACUCCA	5′	23.81%	17	7	0.42	10	0.62	0.68
miR7269	AAUGGAGGAGUUGGAAAGAUU	5′	38.10%	23	11	0.66	12	0.75	0.88
miR7270	CACAAUACUUCCACCAUUGAG	3′	42.86%	12	12	0.72	-	-	-
miR7271	CAAUUCUUCAAUCGCACGUCG	3′	47.62%	11	11	0.66	-	-	-
miR7272	UUCACAUGUUGAAUUACUUGG	5′	33.33%	20	11	0.66	9	0.56	1.18
miR7273	UUGAUAUCAUACUUGAGACUC	5′	33.33%	20	10	0.6	10	0.62	0.97
miR7274	ACUAAAAAAUGGGCAAAUUAG	5′	28.57%	10	3	0.18	7	0.44	0.41
miR7275	AGGUACUAAAUUGAAUAUUGA	3′	23.81%	7	7	0.42	-	-	-
miR7276	AGUGAAUUAAGAACAAACUUU	5′	23.81%	11	5	0.3	-	-	-

RPM, Reads per million. Fold, fold change of TM-1L-A/TM-1L-B.

In total, 487 miRNA precursors were identified in cotton and had an average length of 128.2 nucleotides (Figure S4), while in miRBase (release 19) the average length of 5,166 plant pre-miRNAs was 148.8 nt. The newly identified 93 precursors from the EST and D genome sequences had an average length of 140 nt and were longer than the precursors released in miRBase (131.4 nt). Interestingly, precursors of the 65 novel MIRNAs (148.4 nt) were much longer than the ones of conserved families (124.9 nt).

**Figure 2:**
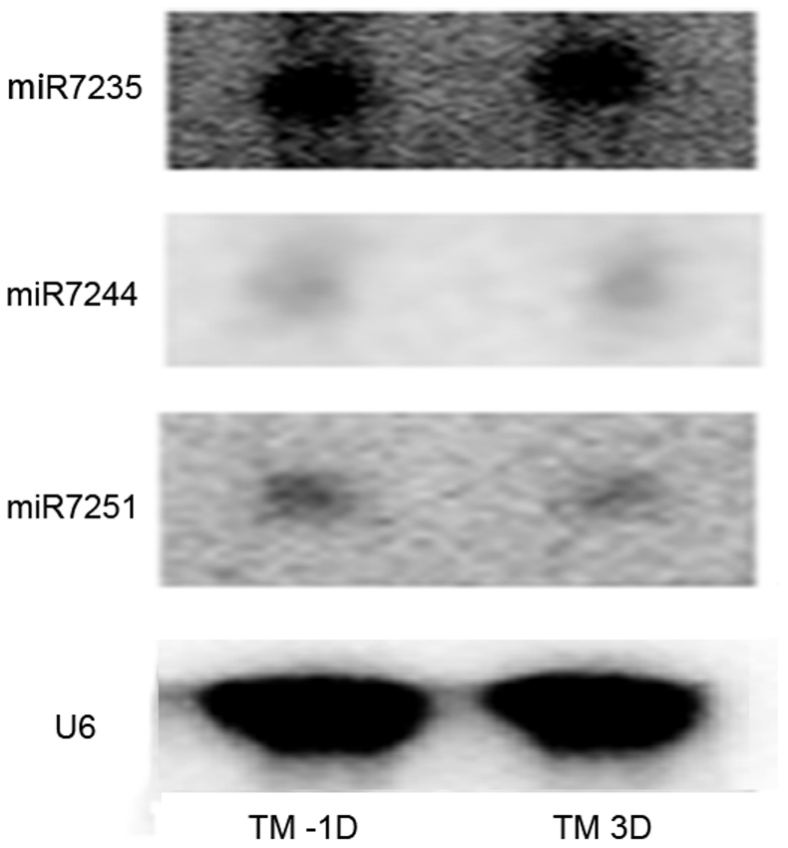
The Northern blot analysis of three novel miRNAs in cotton ovules at −1 and 3 DPA.

As none of the novel miRNA had homologs in miRBase (Release 19), we supposed that they were *Gossypium*-specific, or restricted to closely related species. To validate if they are miRNAs, three of the novel miRNAs, miR7235, miR7244 and miR7251, were randomly selected to conduct the Northern blots. The total RNA from −1DPA and 3DPA ovules was blotted onto Hybond-N^+^ membrane and the Northern blots validated that these three novel miRNAs expressed equally between −1DPA and 3DPA (Figure 2) as the same as the deep sequencing results.

### Targets predicting and validating of cotton miRNAs

**Figure 3:**
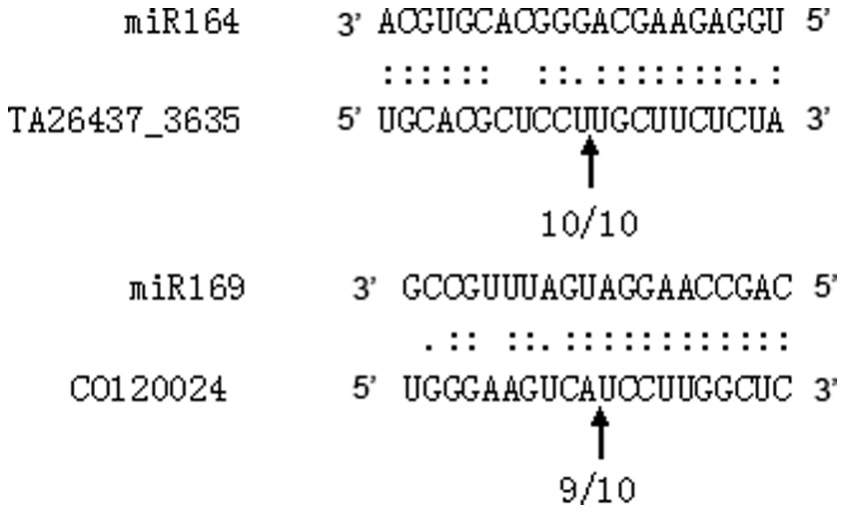
5′ RACE verification of predicted miRNA target genes. The cleavage sites of two selected targets in two miRNA as identified by 5′ RACE analysis. For each miRNA, the miRNA sequence is shown on the top and the target sequence on the bottom. Arrows indicate the cleavage site of the mRNA, and the frequency of clones was shown under the arrow.

In total, 693 EST sequences were obtained and are proposed as candidate target genes (Tables S3 and S4). The 33 known MIRNA families had 300 affiliated target genes, while the 43 novel miRNAs targeted 395 EST sequences. The highly conserved miRNA families have highly conserved target genes, such as miR156 and *SPL* (Table S3). The targets of the novel miRNAs included a much broader range of proteins as compared to those regulated by the miRNAs from the more conserved families (Table S4). To validate the potential targets of miRNAs that might play crucial roles in ovule and fiber early development, two predicted target genes, as an example, were selected and assayed using 5′ RACE. Sequencing of the 5′ RACE products of the two miRNA targets showed that most cleavage sites were mapped to miRNA complementary sequences (Figure 3).

## Discussion

### Two independent libraries validate each other

**Figure 4:**
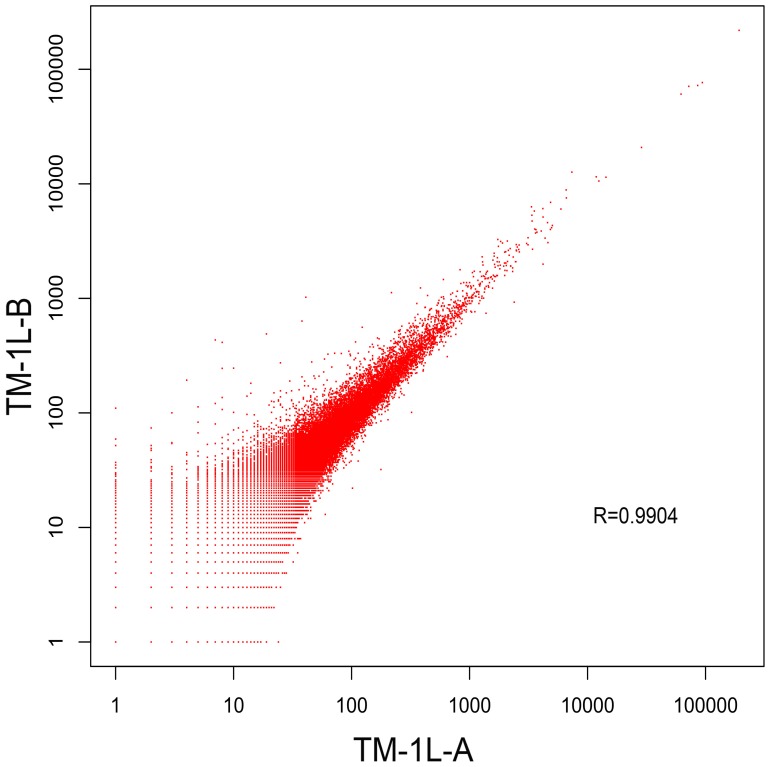
Sequence read counts of small RNAs obtained by sequencing the two libraries. Each point represents a unique small RNA in this log-log scatter plot. The points in red are small RNAs found in both libraries.

Cotton ‘fibers’ are unicellular trichomes derived from epidermal cells of the ovule [9]. The cotton fiber cells undergo a physiological change at −3 DPA when the potential elongation of the cell is determined, although it is not started until anthesis [30], [31]. On the day of anthesis (0 DPA), hemispheroids heave are borne on the surface of cotton ovules, and will grow into fiber cells. The fuzz cells produce a visible morphologic phenotype at 4∼5 DPA. The development of fiber cells is a rapid and continuous progress, and the two libraries produced here could serve as repetitious databases that serve to validate each other. TM-1L-A was pooled from −3, −1, 0 and 1 DPA, and TM-1L-B was pooled from −1, 0, 1, 3 and 5 DPA. The results from the two libraries have little variation across the full dynamic range in the log-log plot (Figure 4). The value of Pearson's correlation coefficient is 0.99. The differences seen between the two databases will reveal distinctions between fiber cell initiation and elongation, as well as fuzz cell initiation processes.

In total, more than thirty-three million reads were obtained from TM-1L-A and TM-1L-B combined (Table 1), which had great potential to dig out novel miRNAs. Among all the reads, the 24 nucleotides reads were in far greater numbers than all others (∼70%; Figure 1), which was consistent with many other reports [32]–[35]. The majority of miRNAs were 21 nt long [36], as the length of the miRNA/miRNA* duplex was determined by the “molecular ruler” property of DCL1 [37]. In miRBase (Release 19.0), 347 mature miRNAs were identified in *A. thaliana*, of which 264 were 21 nt long (76.1%). In rice, the 24 nt long miRNAs were 18.3% of the total miRNAs currently known (132 in 721). The large number of the 24 nt small RNAs found in cotton and described in this study might be small interference RNAs (siRNAs). In *A. thaliana*, the relatively longer small RNAs were prevalently produced in the floral structures (inflorescences) where DCL3, the enzyme responsible for its synthesis, is ten times more abundant relative to its concentration in leaves [38]. The ovules and fibers are contained within flowers and we presume the amount of 24 nt long small RNAs may be due to redundant DCL3 expression in the associated floral organs. The other reports of cotton miRNAs also proposed a similar deduction to explain this phenomenon [32]–[35], [39], [40].

### Identification of novel miRNAs in cotton

Since 2007, almost all of the growth of miRBase has been driven by deep sequencing experiments, which have identified novel miRNAs by the 10s or 100s per experiment [8]. Considering of the complicated nature of plant small RNA, a series of strict filtrations was used to enhance reliability of the result presented here. Currently, no allopolyploid cotton genome sequence was available; therefore ESTs from NCBI and the genome sequence of *G. raimondii* [21], [22], the closest living relative of the progenitor D-genome donor of allotetropolyploid cottons [41], [42] were used as reference sequences to predict the miRNA precursors. To support the positive identification of the mature miRNA sequences they had to be detected in both of the libraries or have more than ten reads in at least one of the library. With the help of high-depth sequencing and the EST and genome sequence of *G. raimondii*, there is a greater potential to find new miRNAs of *Gossypium*. In total, 93 new *MIRNA* genes were identified as eligible candidates for further investigation (Table S1 and S2). A blast search was done to identify their family classification based on conservation which is a powerful indicator of their functional relevance and ancient origins [24]. In total, 28 *MIRNA* genes were found to belong to 10 families (Table S1), which left the other 65 as novel miRNAs (Table S2). In miRBase, besides a few conserved miRNA families, the majority of the families available are restricted to species or subfamily lineages (miRBase, release 19). Not one of the novel miRNAs reported here has a homolog detected in miRBase (Release 19), we proposed them as *Gossypium*-specific or restricted to species that are closely related to cotton. There are 416 precursors predicted according to the sequence of *G. raimondii*. No significant regularities of distribution were found, but there was a specific genomic region on Chr. D5 where the MIRNAs clustered (Figure S5).

### Function of miRNAs in fiber and seed initial development

Cotton fibers share many similarities with *Arabidopsis* leaf trichomes, which could serve as a model for elucidating the genetic mechanisms that control the development of cotton fiber and seeds [10]. MiRNAs have been shown to play an important role in cotton fiber and seed development. MiR166 was found mostly accumulated miRNAs in cotton young ovules and fibers with more than 75,000 reads between the two libraries used here (Table 2). The targets of miR166 were predicted to express Class III HD-ZIP family of transcription factors with functions of organ polarity and morphogenesis [6]. MiR166 was also shown to be involved in the distribution of trichomes [16]. Another miRNA shown to control trichome development in *Arabidopsis* was miR156, and its target, *SPL*9, was shown to define function in an endogenous flowering pathway; this finding established a direct link between developmental programming and trichome distribution [15]. Thousands of miR156/157 was accumulated during the initial development of the cotton fibers and seeds, and fifteen targets of miR156/157 were predicted to express SPL transcription factors.

MiR172 also had a significant expression with more than sixty thousand reads between each of the two libraries (Table 2). MiR172 targets the AP2 transcription factor in *Arabidopsis* and maize, and it have been validated miR172 together with miR156 regulate development transitions in complementary patterns and guide various aspects of reproductive development [11]–[14]. The expression levels of *SPL9* increase in conjunction with inflorescence development, which reflects a decrease in miR156 [15]. MiR156 regulates the expression of miR172 *via* SPL9 which, redundantly with SPL10, directly promotes the transcription of *miR172b* [12]. The cotton ovules have high expression levels of miR172 and have relatively low levels of miR156/157, which was consisted with their conversely regulation patterns [6], implying they play an important role in the development of this organ.

Auxin plays a crucial role in seed development [18] and cotton fiber initiation [17]. Both miR167 and miR160 could target *ARF* mRNAs to regulate the accumulation of the auxin, and in *Arabidopsis*, miR160 targets *ARF10*, *ARF16* and *ARF17*, while miR167 targets *ARF6*, *ARF8* and *IAR3*. Plants expressing a miRNA-resistant version of *ARF17* have increased *ARF17* mRNA levels and reduced accumulation of auxin-inducible *GH3*-like mRNAs, which encode auxin-conjugating proteins [43]. However, *OsGH3-2* was positively regulated by ARF8 [44]. Meanwhile, another target of miR167, *IAA-Ala Resistant3* (*IAR3*), encode an enzyme hydrolyzing an inactive form of auxin (IAA-alanine) and releases bioactive auxin (IAA) [45]. In the libraries of TM-1L-A and TM-1L-B, the expression levels of miR167 were 33-fold and five-fold higher than miR160, as miR160 was notably upregulated in TM-1L-B (Table 2). These results were consistent with the high auxin accumulation during fiber initiation, and endogenous IAA levels in ovules were reduced continuously from 1 DPA to 3 DPA [17]. MiR167 was also shown to be essential for the fertility of ovules and the relatively high level of IAA was imperative for proper zygote polarity and development [19], [46].

Besides miR160, miR394, miR397, miR398, miR482 and miR2111 were also significantly upregulated in TM-1L-B compared to TM-1L-A, suggesting that the miRNA population changed during the early stages of cotton ovule development. MiR397 targets genes encoding a series of laccase and steroid-binding protein by cleavage or translation repression. The targets of miR398 were *Cu/Zn SUPEROXIDE DISMUTASE* (*SOD*) mRNAs. The targets of miR394, miR482 and miR2111 were genes encoding enzyme or other protein. All of these target genes are known involving in the regulation of metabolic processes. A function for miRNAs as safeguards against unwanted gene expression is a common theme in eukaryotes [6], [7], [47]. The increase in the six miRNAs in TM-1L-B seemed to suggest it was no longer necessary to preserve the same level of the gene expression after fiber initiation as the cell fate had been determined.

All of the 43 novel miRNA described here are expressed equally between the TM-1L-A and TM-1L-B libraries (Table 3). MiR7234 was accumulated in high levels with more than twenty thousand reads and targeted two ESTs, which expressed starch synthase and retrotransposon. MiR7235, miR7236 and miR7237 were detected with more than one thousand reads in each library. However, the majority of the novel miRNAs (26, 60.5%) were detected less than 50 times. Northern blots validated miR7235, miR7244 and miR7251 expressed equally between −1DPA and 3DPA (Figure 2). Combining Northern blots results and sequencing data, we infer that the expression level of these three novel miRNAs change little during the early stage of fiber development. Unlike the targets of conserved miRNA families that have a tendency to be transcription factors, less conserved miRNAs target genes that have more flexibility cover a large-scale of aspect of functions in cotton, such as metabolism, transcription factors, structure protein, signals transduction and so on which play essential roles in seed set development [18].

## Supporting Information

Figure S1 **Secondary structure of the precursors of the new members of the known families.**
(TIF)

Figure S2 **Secondary structure of the precursors of the novel miRNA.**
(TIF)

Figure S3 **Analyses of nuclleotide bias at each position along the novel miRNAs.**
(TIF)

Figure S4 **Length distribution of MIRNA precursors in plant.**
(TIF)

Figure S5 **MIRNAs distribution on the 13 assemble** ***G. raimondii*** **chromosomes.**
(TIF)

Table S1 **New members of known families of microRNAs.**
(XLS)

Table S2 **Novel miRNAs identified in the present report.**
(XLS)

Table S3 **Targets of known cotton miRNAs.**
(XLS)

Table S4 **Targets of novel miRNAs identified in the present report.**
(XLS)

Table S5 **Primers and probes used in this study.**
(XLSX)
