# Supplementary figures and images for: Genome-Wide Analysis of Small RNA and Novel MicroRNA Discovery during Fiber and Seed Initial Development in Gossypium hirsutum. L
Source: PLoS One. 2013 Jul 29;8(7):e69743. doi: 10.1371/journal.pone.0069743 (PMC3726788; doi:10.1371/journal.pone.0069743)

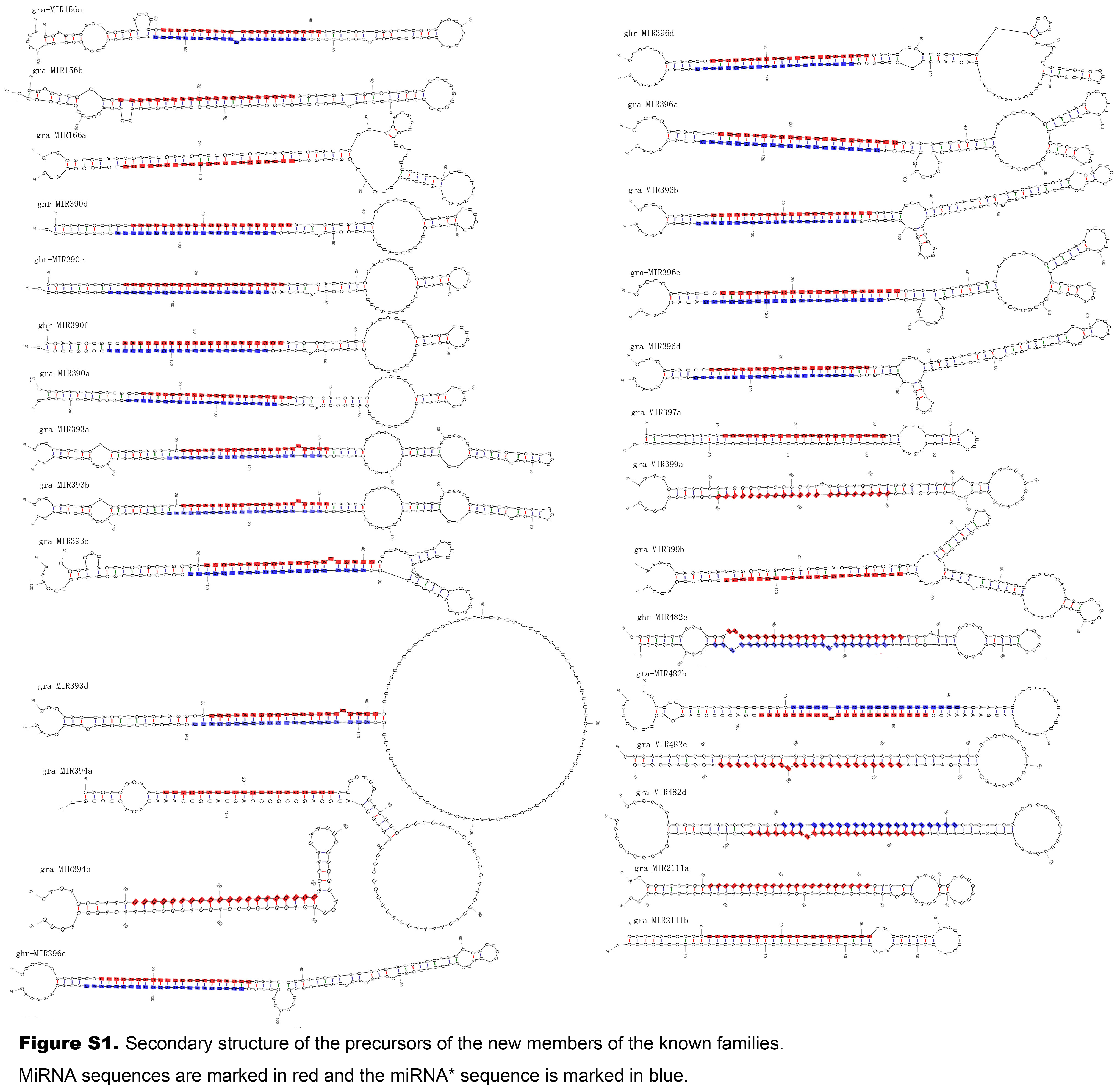

Supplement: Figure S1 — Secondary structure of the precursors of the new members of the known families. (TIF) [file pone.0069743.s001.tif]

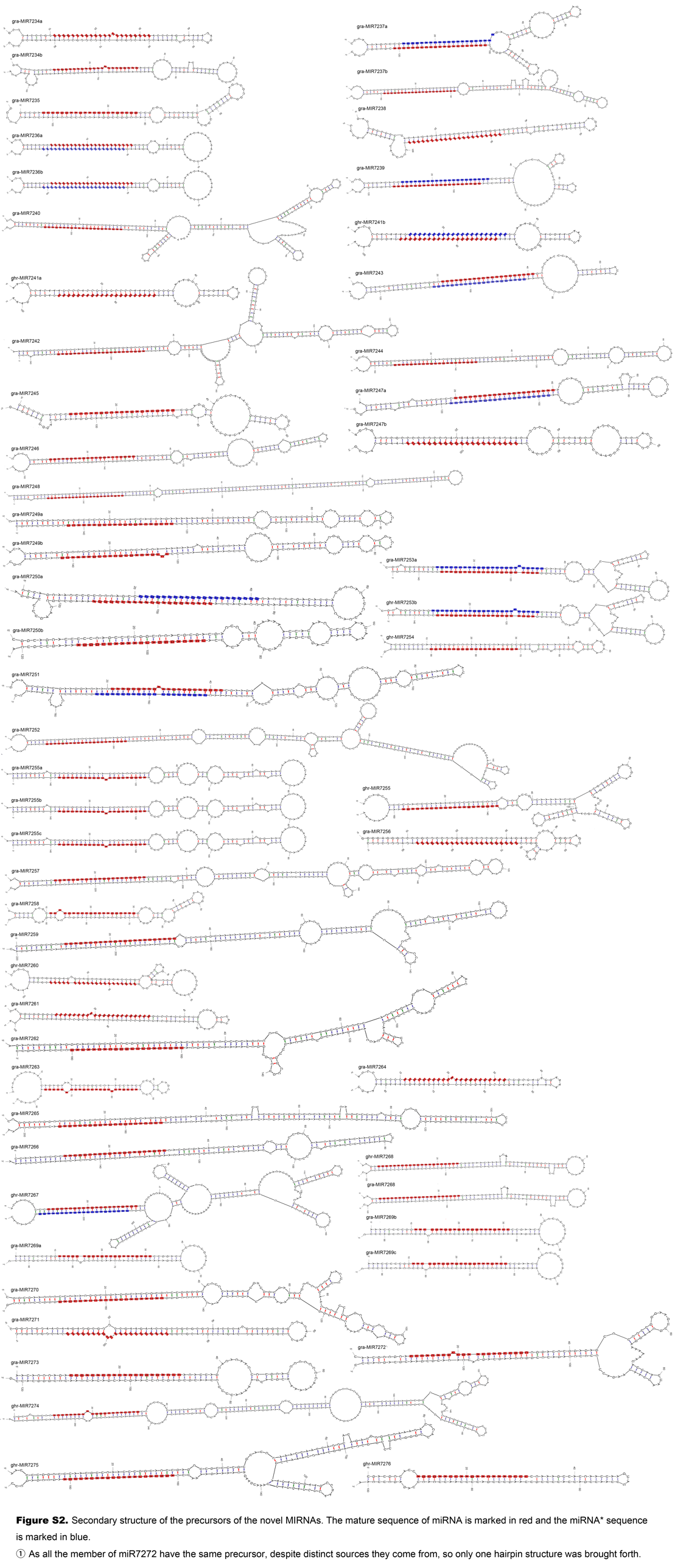

Supplement: Figure S2 — Secondary structure of the precursors of the novel miRNA. (TIF) [file pone.0069743.s002.tif]

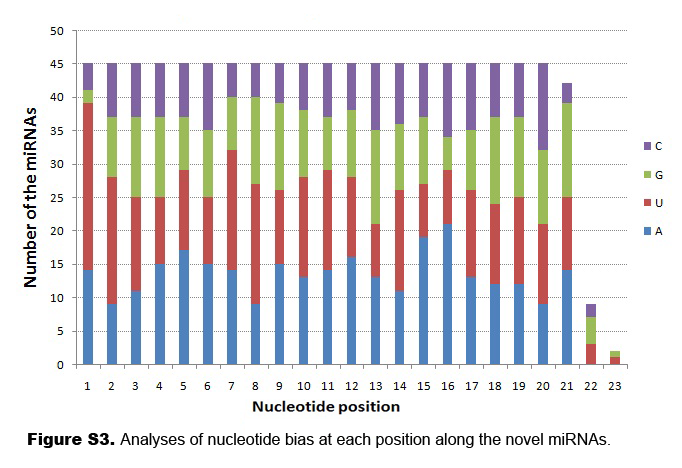

Supplement: Figure S3 — Analyses of nuclleotide bias at each position along the novel miRNAs. (TIF) [file pone.0069743.s003.tif]

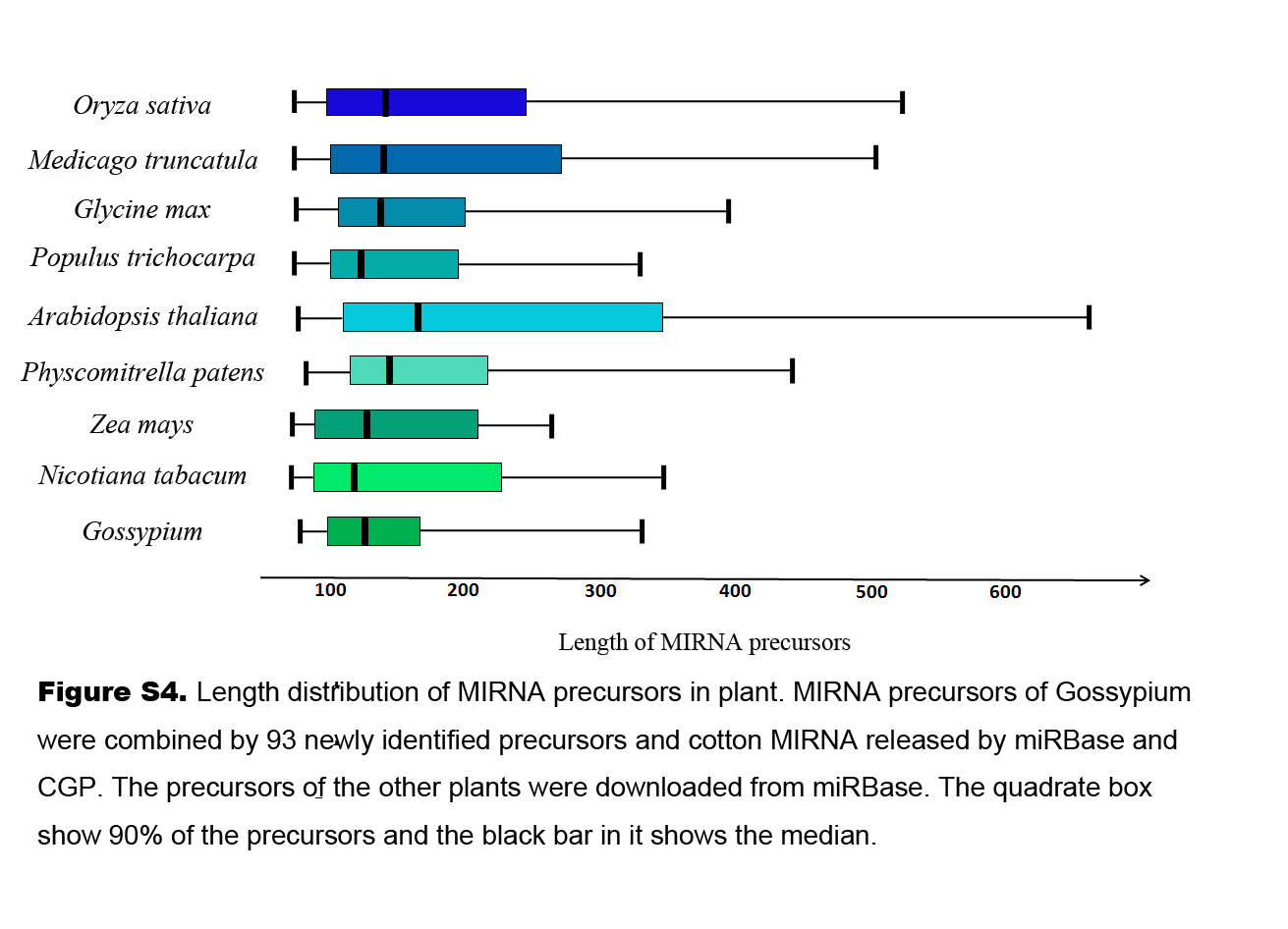

Supplement: Figure S4 — Length distribution of MIRNA precursors in plant. (TIF) [file pone.0069743.s004.tif]

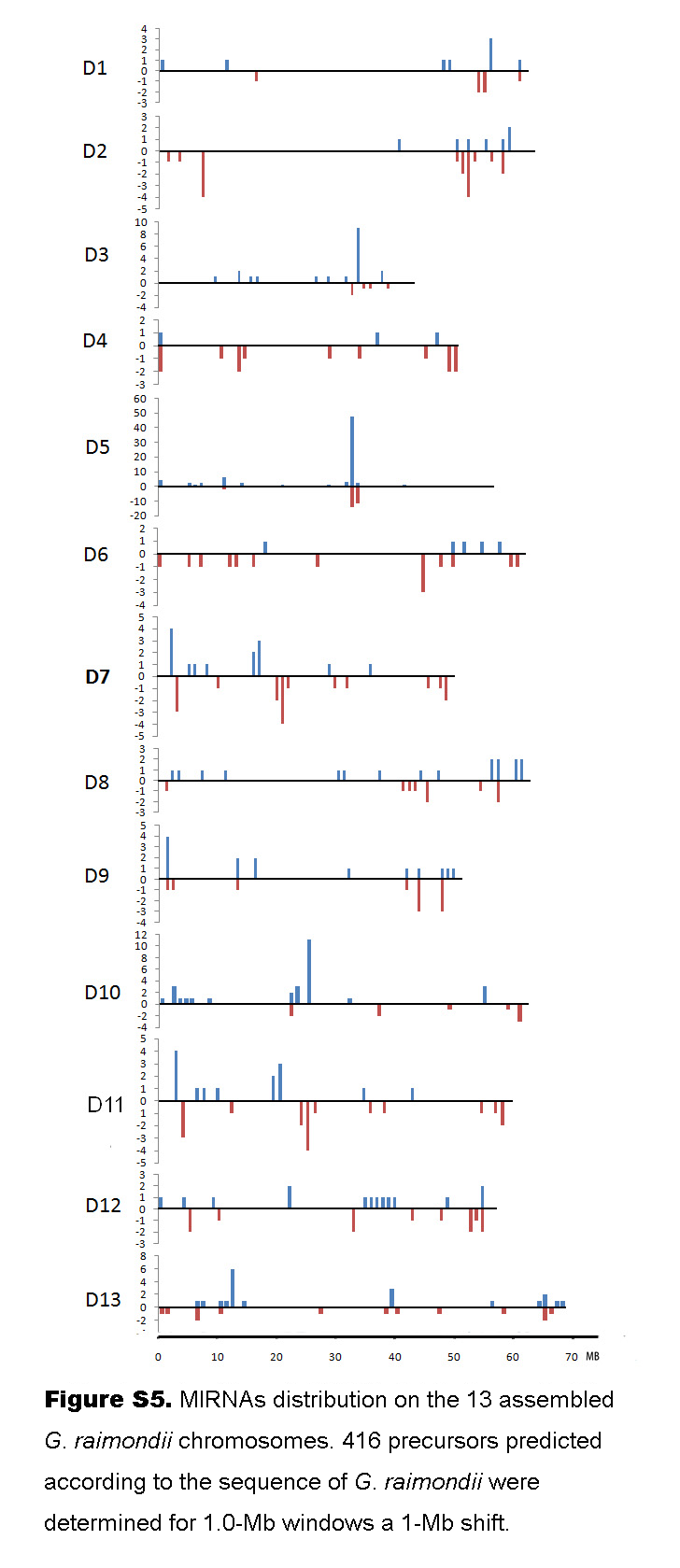

Supplement: Figure S5 — MIRNAs distribution on the 13 assemble G. raimondii chromosomes. (TIF) [file pone.0069743.s005.tif]
